# Long-Term Parasitological Clearance and Cardiac Progression in *Trypanosoma cruzi* TcI: A Phase-Stratified Cohort from Colombia

**DOI:** 10.3390/tropicalmed11020059

**Published:** 2026-02-19

**Authors:** Mario J. Olivera, Adriana Arévalo, Alejandro Marcel Hasslocher-Moreno

**Affiliations:** 1 Grupo de Parasitología, Instituto Nacional de Salud, Bogotá 111321, Colombia; aarevalo@ins.gov.co; 2 Evandro Chagas National Institute of Infectious Diseases, Oswaldo Cruz Foundation, Rio de Janeiro 21040-360, Brazil; alejandro.hasslocher@gmail.com

**Keywords:** Chagas disease, *Trypanosoma cruzi*, genotype, disease progression, Colombia

## Abstract

Clinical trajectories following *Trypanosoma cruzi* infection are heterogeneous, and the determinants of post-treatment parasitological dynamics and cardiac progression remain incompletely characterized, particularly in TcI-predominant regions. This study assessed, in both the acute phase and the indeterminate chronic form, the association between TcI infection and molecular clearance kinetics, cardiac progression, and the prognostic value of early molecular response. An ambispective cohort in Colombia included patients with acute or indeterminate chronic infection followed between 2000 and 2023. Sustained clearance was defined as two consecutive negative quantitative polymerase chain reaction results obtained at separate visits. Time-to-event analyses were conducted using Kaplan–Meier curves and Cox proportional hazards models. TcI infection was consistently associated with delayed molecular clearance in both clinical presentations. Although long-term clearance was achieved in most patients, TcI infection was independently associated with a higher risk of cardiac progression. In contrast, quantitative polymerase chain reaction negativity at 12 months was associated with reduced subsequent progression risk, indicating that sustained molecular response is a clinically meaningful prognostic marker. Collectively, these findings support the incorporation of early molecular monitoring into risk-stratified follow-up strategies in TcI-predominant settings and reinforce the need for phase-specific clinical management approaches.

## 1. Introduction

Clinical outcomes after *Trypanosoma cruzi* infection vary widely across individuals and endemic regions, ranging from lifelong indeterminate infection to progressive cardiac disease associated with substantial morbidity and premature mortality [1,2]. This heterogeneity is not fully explained by host susceptibility or access to care [3]. In endemic settings, clinical expression reflects interactions among parasite genetic diversity, transmission ecology, exposure intensity, and environmental and social context [4]. In regions where Discrete Typing Unit (DTU) TcI predominates, these interactions are particularly consequential, as long-standing assumptions of uniformly benign clinical behavior have been increasingly questioned [5].

Chagas disease remains a major public health challenge in Latin America and an expanding concern in non-endemic regions due to population mobility [6]. More than six million individuals are estimated to be infected worldwide, with chronic Chagas cardiomyopathy accounting for most disease-related morbidity and mortality through conduction abnormalities, ventricular arrhythmias, heart failure, stroke, and sudden death [7,8]. Despite advances in diagnostics and etiological therapy, long-term prognosis and risk of cardiac progression remain difficult to predict at the individual level, particularly in TcI-predominant regions where clinical trajectories are heterogeneous and strongly shaped by ecological context [9].

A defining feature of *T. cruzi* is its extensive genetic diversity, currently classified into seven DTUs TcI to TcVI and TcBat. These DTUs differ in ecological distribution, transmission cycles, tissue tropism, and biological behavior, with context-dependent associations with clinical expression and therapeutic response [10]. Comparative evidence across endemic regions supports geographically structured DTU–phenotype relationships shaped by eco-epidemiological conditions, including vector and reservoir ecology, route of infection, inoculum size, and transmission intensity [11,12,13]. According to this body of work, universal or deterministic genotype–phenotype attribution is not justified, underscoring the need for region-specific and phase-specific investigation.

In the Southern Cone, where DTUs TcII, TcV, and TcVI predominate, multiple cohorts have linked these lineages to severe chronic Chagas cardiomyopathy and conduction system disease [14,15,16,17]. In contrast, Andean and Amazonian regions, including Colombia and Venezuela, are predominantly affected by TcI, which has historically been associated with indeterminate or milder chronic forms [18,19]. In recent years, this interpretation has been reconsidered. Acute Chagas disease in these regions has been reported in ecological contexts characterized by high exposure intensity and marked early parasitemia, conditions that are frequently associated with oral transmission and are linked to severe clinical presentations, including acute myocarditis [20,21]. Emerging longitudinal evidence from TcI-predominant settings describes clinically relevant cardiac progression under specific ecological and exposure conditions [22].

Within TcI, subgenotypes associated with domestic and sylvatic transmission cycles, commonly referred to as TcIDOM and TcISYL, display distinct epidemiological distributions and parasitological profiles. Observations from Colombian cohorts describe higher frequencies of TcIDOM among chronic patients and higher baseline parasite burdens compared with TcISYL, supporting an eco-epidemiological gradient relevant to long-term outcomes rather than a dichotomous genotype effect [22]. In acute clusters linked to oral transmission, high inoculum exposure and intense early parasitemia complicate attribution of early disease severity to parasite lineage independently of route and dose [23].

In this context, the present study aimed to evaluate, separately in the acute clinical phase and the indeterminate chronic form, the association between DTU TcI infection and post-treatment parasitological clearance kinetics, long-term cardiac progression, and the prognostic relevance of early molecular response in TcI-predominant settings.

## 2. Materials and Methods

### 2.1. Study Design and Setting

An ambispective observational cohort study was conducted at the Instituto Nacional de Salud in Bogotá, Colombia. Patients evaluated between June 2000 and December 2023 were eligible for inclusion. Because acute and indeterminate chronic *T. cruzi* infection differ in biological behavior, exposure structure, and clinical trajectories, all analyses were prespecified and stratified by clinical phase. Reporting followed the Strengthening Reporting of Observational Studies in Epidemiology guidelines. Diagnostic protocols and electrocardiographic interpretation criteria were harmonized across study periods using standardized institutional guidelines to ensure longitudinal consistency.

### 2.2. Study Population and Eligibility Criteria

Participants were eligible if they had confirmed *T. cruzi* infection and met criteria for inclusion in either the acute or indeterminate chronic cohort. Patients were enrolled through multiple outbreak investigations conducted within routine public health surveillance, with the number of participants contributed by each outbreak ranging from 1 to 27 individuals. Inclusion required phase-appropriate laboratory confirmation, molecular confirmation by quantitative polymerase chain reaction (qPCR) with parasite genotyping, and availability of at least two paired 12-lead electrocardiograms (ECGs) to allow longitudinal cardiac assessment.

For the chronic cohort, patients were required to be in the indeterminate form at baseline. Individuals with established cardiac disease at enrollment were excluded. Participants were excluded if follow-up was shorter than four years, molecular or ECG data were incomplete, or etiological therapy with benznidazole or nifurtimox was discontinued permanently before completing thirty days. Temporary treatment interruptions under medical supervision were permitted and documented.

To preserve interpretability of genotype-specific associations, infections with mixed DTUs involving TcI in combination with other DTUs were excluded. Mixed infections not involving TcI were retained and analyzed within the non-TcI reference group. Individual non-TcI DTUs were grouped a priori due to limited counts to avoid unstable estimates and post hoc comparisons.

### 2.3. Clinical Definitions and Outcomes

Acute infection was defined by direct parasitological detection of *T. cruzi* in peripheral blood using microhematocrit or Strout methods [24]. Indeterminate chronic infection was defined by positive serology using enzyme-linked immunosorbent assay (ELISA) and indirect immunofluorescence assay (IFA), absence of cardiac involvement, and normal baseline findings on conventional 12-lead ECG and chest radiography [25]. Gastrointestinal studies were not performed in the absence of symptoms.

Two outcomes were predefined. The primary outcome was parasitological clearance following etiological therapy, defined as sustained qPCR negativization, operationalized as two consecutive negative qPCR results obtained on different follow-up visits. No fixed minimum interval between negative results was prespecified, reflecting routine clinical follow-up practices and variability in visit schedules.

The secondary outcome was cardiac progression, defined by the appearance of at least two new ECG abnormalities, including complete right bundle branch block, frequent ventricular extrasystoles, second- or third-degree atrioventricular block, sinus bradycardia below fifty beats per minute, atrial fibrillation, or ST–T segment abnormalities.

A composite cardiovascular outcome including heart failure, stroke, pacemaker or implantable cardioverter defibrillator implantation, and death was recorded descriptively. Transition from acute to chronic infection was defined by persistent seropositivity on two independent assays (ELISA and IFA). Serological results were analyzed longitudinally as supportive information and did not override molecular results when qPCR remained positive.

### 2.4. Etiological Treatment and Adherence

Etiological treatment with benznidazole (5–10 mg/kg/day) or nifurtimox (8–10 mg/kg/day) was initiated according to national drug availability and standard clinical practice [25,26]. Therapy was administered on an outpatient basis under standardized clinical supervision.

Follow-up was conducted on days 15, 30, and 60 for all participants. Additional visits were scheduled as clinically indicated to evaluate adherence, adverse events, or emerging cardiovascular symptoms. Treatment duration, temporary interruptions, and adverse events were systematically recorded. Adherence was assessed by pill count and patient self-report.

### 2.5. Molecular and Serological Assessments

Peripheral blood samples were obtained at baseline (prior to etiological therapy) and at predefined follow-up visits at 6, 12, and 24 months, and annually thereafter. Additional samples were collected when clinically indicated. qPCR for *T. cruzi* DNA followed validated institutional protocols, with parasite load expressed as parasite equivalents/mL [5,24]. Parasite genotyping employed established molecular markers to classify DTUs and TcI subgenotypes [5,24].

Serological monitoring using ELISA and IFA was performed at the same time points as qPCR assessments to ensure paired molecular–serological evaluation [24]. Antibody titers were analyzed longitudinally to characterize serological trajectories over time.

### 2.6. Transmission Route Considerations

All acute cases included in the study were linked to documented oral transmission outbreaks, ensuring homogeneity of transmission route within the acute cohort. In contrast, transmission route in chronic infection could not be reliably determined retrospectively due to long latency and heterogeneous exposure histories and was therefore not included as a covariate in multivariable analyses.

### 2.7. Follow-Up Schedule

Follow-up time was calculated from the date of the baseline 12-lead ECG to the occurrence of study outcomes or the last recorded clinical visit before administrative censoring in December 2023. At baseline, all participants underwent a standardized clinical and epidemiological assessment, including medical history, cardiovascular focused physical examination, chest radiography, ECG, qPCR, and serological testing.

Participants were followed annually with repeat ECG and molecular and serological assessments. Additional clinical and diagnostic evaluations were performed when cardiovascular events were identified or when new symptoms prompted intensified follow-up according to standard clinical practice.

### 2.8. Statistical Analysis

Baseline characteristics were summarized using descriptive statistics. Continuous variables were reported as mean with standard deviation or median with interquartile range (IQR), according to their distribution, while categorical variables were expressed as frequencies and percentages. Baseline acute clinical manifestations were described descriptively and were not analyzed as outcomes.

For the primary outcome, parasitological clearance was analyzed using time-to-event methods. Kaplan–Meier analyses were used to estimate time to sustained qPCR negativization, and differences between DTU groups were evaluated using log-rank tests. Cox proportional hazards models were used to estimate associations between DTU TcI infection and the rate of sustained qPCR negativization, expressed as hazard ratios (HRs) with 95% confidence intervals (CIs).

Time-to-event analyses were conducted for cardiac progression using Kaplan–Meier methods and Cox proportional hazards models. Associations between DTU TcI infection and cardiac progression were estimated as HRs with 95% CIs, using non-TcI DTUs as the reference group. The non-TcI category included TcII, TcIII, TcIV, TcVI, and mixed non-TcI infections (TcII–TcIV, TcII–TcVI, TcIII–TcVI). Individual non-TcI DTUs were grouped a priori due to limited counts, and infections with mixed TcI and non-TcI DTUs were excluded to preserve interpretability of genotype-specific associations.

Multivariable models were adjusted for age, sex, baseline parasitemia, duration of residence in endemic areas, and comorbidities including hypertension, diabetes mellitus, and dyslipidemia.. Proportional hazards assumptions were evaluated using Schoenfeld residuals, and collinearity among covariates was assessed using variance inflation factors. For analyses of cardiac progression, the prognostic value of sustained qPCR negativization at 12 months post-treatment was evaluated in Cox models.

To account for death as a competing event in analyses of cardiac progression, prespecified sensitivity analyses were conducted using Fine–Gray subdistribution hazard models. Subdistribution hazard ratios with 95% CIs were estimated to evaluate the association between DTU TcI infection and the cumulative incidence of cardiac progression in the presence of competing mortality. Gray’s test was used to compare cumulative incidence functions between DTU groups.

Prespecified sensitivity analyses included alternative definitions of sustained qPCR negativization requiring a minimum interval (≥30 days) between consecutive negative results, as well as stratified analyses by etiological drug, TcI subgenotype, and baseline parasitemia tertiles.

All analyses were performed using R software (R Foundation for Statistical Computing, Vienna, Austria). Statistical significance was defined as a two-sided *p* value below 0.05.

### 2.9. Ethical Considerations

The study was approved by the Technical Research Committee and the Research Ethics Board of the Instituto Nacional de Salud in Bogota, Colombia (protocol CTIN014-11). Participation was voluntary, and written informed consent was obtained from all participants for the use of clinical and laboratory data. All data were anonymized and analyzed using coded identifiers to ensure confidentiality.

## 3. Results

### 3.1. Baseline Clinical and Epidemiological Characteristics

Between 2000 and 2023, a total of 220 patients with confirmed *T. cruzi* infection were included in the study. Of these, 101 were diagnosed during the acute phase, all linked to documented oral transmission outbreaks, and 119 were enrolled in the indeterminate chronic form. Median age at baseline was 32 years (IQR 20–49) in the acute cohort and 47 years (IQR 41–51) in the chronic cohort. Male sex predominated in acute infection (62.8%), whereas females were more frequent in chronic infection (61.3%).

Comorbidities were more prevalent among patients with chronic infection, including hypertension in 47 cases (39.4%), dyslipidemia in 34 cases (28.5%), and diabetes mellitus in 31 cases (26.1%). Prolonged residence in endemic areas exceeding ten years was also more frequent in the chronic cohort with 83 cases (69.7%) compared with 42 cases (41.6%) in the acute cohort.

At diagnosis, ECG abnormalities were identified in 65 cases (64.3%) in the acute cohort. The most frequent findings included nonspecific repolarization changes in 29 cases (28.7%), sinus tachycardia in 12 cases (11.9%), right bundle branch block in 9 cases (8.9%), atrioventricular conduction disturbances in 7 cases (6.9%), and ST–T segment abnormalities in 2 cases (2.0%). At 12 months post-treatment, complete resolution of ECG alterations was observed in 42 cases (64.6%). Among the remaining 23 cases (35.4%), persistence of the initial abnormalities without interval change was observed in 15 cases, while 8 cases developed new or progressive findings, most commonly new conduction disturbances in 5 cases and persistent repolarization changes evolving to right bundle branch block in 3 cases. No patient developed complex ventricular arrhythmias during this period.

Acute myocarditis was diagnosed in 43 cases (42.5%) based on compatible clinical presentation and ECG findings, with or without biomarker elevation, according to institutional criteria. Among patients with myocarditis, 26 cases (60.5%) achieved complete ECG normalization at 12 months, whereas 17 cases (39.5%) exhibited persistent or new abnormalities as described above. Myocarditis occurred more frequently among patients infected with DTU TcI.

DTU TcI was the predominant genotype in both cohorts, identified in 79 acute cases (78.2%) and 81 chronic cases (68.1%). Non-TcI infections accounted for 22 cases (21.8%) in the acute cohort and 38 cases (31.9%) in the indeterminate chronic cohort. This non-TcI group was heterogeneous and included TcII in 6 acute and 10 chronic cases, TcIII in 4 acute and 7 chronic cases, TcIV in 7 acute and 9 chronic cases, TcVI in 3 acute and 6 chronic cases, and mixed non-TcI infections in 2 acute and 6 chronic cases, accounting for all non-TcI cases in each cohort, with no single lineage predominating. Individual non-TcI DTUs were not analyzed separately due to the small and uneven subgroup sizes, which could lead to unstable estimates and clinically uninterpretable post hoc comparisons. Within TcI infections, TcISYL predominated in the acute cohort, whereas TcIDOM was more frequent in the chronic cohort.

**Figure 1:**
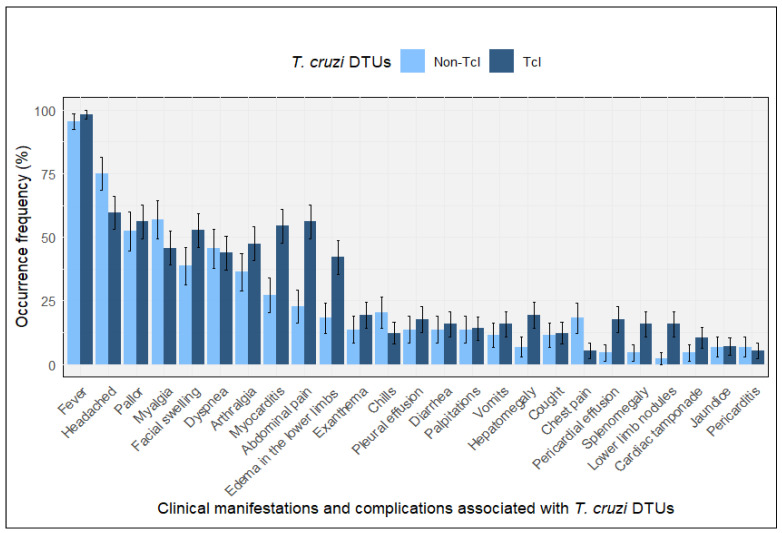
Baseline acute clinical manifestations by DTU group. Bars represent the proportion of acute cases reporting each manifestation at presentation, stratified by DTU group (TcI, *n* = 67; non-TcI, *n* = 34). Data are presented for descriptive purposes only; no hypothesis testing was performed. Error bars represent 95% binomial confidence intervals.

Baseline acute clinical manifestations were described according to DTU group to provide clinical context for the outbreak-linked acute cohort. Across DTU groups, the most frequently reported manifestations at presentation were fever, headache, myalgia, and asthenia, followed by gastrointestinal symptoms and cardiac involvement, including myocarditis and arrhythmias. The distribution of acute manifestations by DTU group is shown in Figure 1. As prespecified, acute clinical manifestations were presented for descriptive purposes only; no hypothesis testing was performed, and these manifestations were not considered study outcomes.

Baseline characteristics are summarized in Table 1. No inferential comparisons were performed between cohorts due to distinct epidemiological contexts.

### 3.2. Etiological Treatment, Adherence, and Adverse Events

All patients completed etiological therapy with benznidazole or nifurtimox, with treatment duration ranging from 60 to 82 days. Benznidazole was administered in 142 cases (64.5%) and nifurtimox in 78 cases (35.5%). Temporary treatment interruptions due to moderate adverse events occurred in 10 cases (4.5%); therapy was resumed in all cases, and no permanent discontinuations were recorded.

Among patients treated with benznidazole, at least one adverse event was reported in 122 cases (85.9%). The most common dermatological manifestations included rash in 48 cases (33.8%), pruritus in 41 cases (28.9%), and urticaria in 10 cases (7.0%). Gastrointestinal symptoms were also frequent, including epigastric pain in 34 cases (23.9%), abdominal bloating in 30 cases (21.1%), and nausea in 27 cases (19.0%). Angioedema occurred in 9 cases (6.3%) and photosensitivity in 6 cases (4.2%).

In the nifurtimox group, adverse events were reported in 63 cases (80.8%), predominantly gastrointestinal and neurological symptoms. The most frequent included epigastric pain in 16 cases (20.5%), nausea in 13 cases (16.7%), sleep disturbances in 12 cases (15.4%), loss of appetite in 12 cases (15.4%), and transient memory impairment in 10 cases (12.8%).

### 3.3. Primary Outcome: Parasitological Clearance by qPCR

#### 3.3.1. Acute Cohort

**Figure 2:**
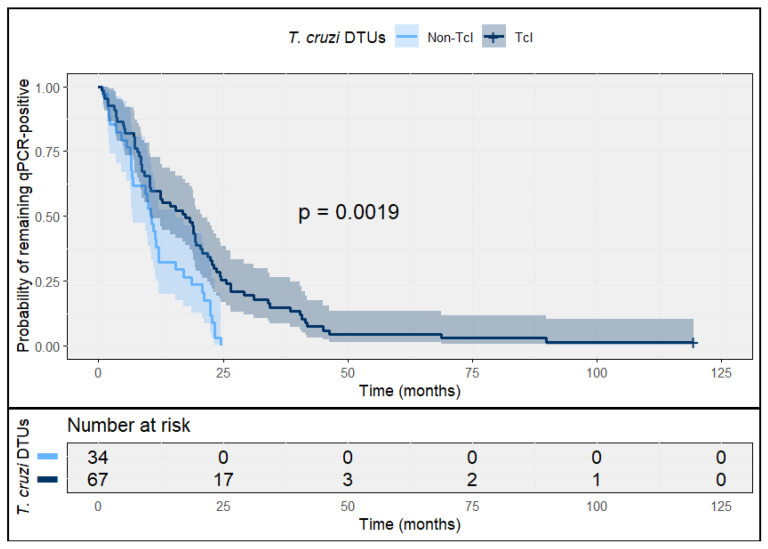
Time to sustained qPCR negativization after etiological treatment in acute *T. cruzi* infection, stratified by DTU. Kaplan–Meier curves depict the probability of remaining qPCR-positive during follow-up among patients with acute *T. cruzi* infection, comparing TcI and non-TcI DTUs. Sustained qPCR negativization was defined as two consecutive negative qPCR results obtained at separate follow-up visits. Shaded areas represent 95% confidence intervals; numbers at risk are shown below the plot.

At 12 months post-treatment, persistent qPCR positivity occurred predominantly among patients infected with DTU TcI, whereas non-TcI infections reached qPCR negativization earlier. Kaplan–Meier analyses for time to qPCR negativization indicated a statistically significantly higher probability of remaining qPCR-positive among TcI infections (log-rank *p* = 0.0019) during early follow-up (Figure 2).

In Cox proportional hazards models for time to sustained qPCR negativization, TcI infection was associated with a lower rate of molecular clearance compared with non-TcI DTUs (HR 0.62; 95% CI 0.41–0.93; log-rank *p* = 0.0001), indicating slower clearance kinetics among TcI infections. Despite these differences in early clearance kinetics, sustained qPCR negativization was observed in all patients during long-term follow-up, and by 120 months no patient had detectable parasitemia by qPCR (Figure 2).

In prespecified sensitivity analyses, effect estimates were directionally similar after adjustment for baseline parasitemia (log10), etiological drug, age, and sex (adjusted HR 0.66; 95% CI 0.43–0.99). No evidence of effect modification by etiological drug was observed (interaction *p* > 0.10).

#### 3.3.2. Chronic Cohort

**Figure 3:**
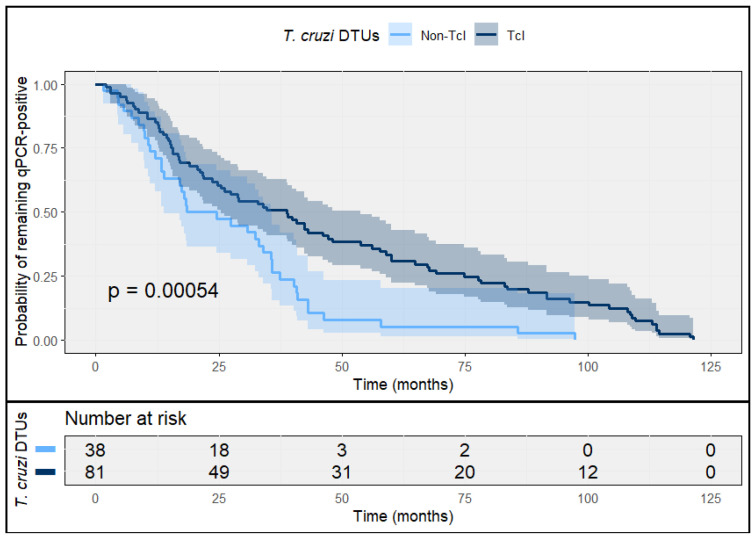
Time to sustained qPCR negativization after etiological treatment in indeterminate chronic *T. cruzi* infection, stratified by DTU. Kaplan–Meier curves depict the probability of remaining qPCR-positive during follow-up among patients with indeterminate chronic *T. cruzi* infection, comparing TcI and non-TcI DTUs. Sustained qPCR negativization was defined as two consecutive negative qPCR results obtained at separate follow-up visits. Shaded areas represent 95% confidence intervals; numbers at risk are shown below the plot.

In the indeterminate chronic cohort, molecular clearance occurred more slowly than in acute infection. Kaplan–Meier analyses for time to qPCR negativization indicated a persistently higher probability of remaining qPCR-positive among TcI infections compared with non-TcI infections during early follow-up, consistent with delayed clearance kinetics in TcI-predominant settings (Figure 3).

In Cox proportional hazards models, TcI infection was associated with a lower rate of sustained qPCR negativization compared with non-TcI DTUs (HR 0.46; 95% CI 0.30–0.70; log-rank *p* = 0.0002), indicating slower molecular clearance among TcI infections. Despite these differences in clearance kinetics, sustained qPCR negativization was observed in nearly all patients during extended follow-up, and by 120 months qPCR positivity was uncommon across DTU groups (Figure 3).

In prespecified sensitivity analyses, the association between TcI infection and slower molecular clearance was consistent across strata of baseline parasitemia, etiological drug, and model specifications. When baseline parasitemia was categorized into tertiles, lower rates of qPCR negativization among TcI infections were observed across all tertiles, with greater separation in the highest parasitemia tertile (adjusted HR 0.38; 95% CI 0.22–0.65) and attenuation in the lowest tertile (adjusted HR 0.62; 95% CI 0.41–0.94).

Clearance kinetics were comparable between patients treated with benznidazole and nifurtimox, with no evidence of effect modification by drug type (interaction *p* > 0.10). Results were also robust to alternative model specifications excluding mixed non-TcI infections and to models defining molecular clearance using sustained qPCR negativization criteria based on two consecutive negative results.

### 3.4. Secondary Outcome: Cardiac Progression

#### 3.4.1. Acute Cohort

During a median follow-up of 168 months (IQR 120–180), 23 patients (22.8%) progressed to chronic cardiac involvement after transition to chronic infection. The cumulative incidence of cardiac progression was statistically significantly higher among TcI infections (28.4%; 95% CI 19.4–39.5) than among non-TcI infections (11.8%; 95% CI 2.1–23.4).

**Figure 4:**
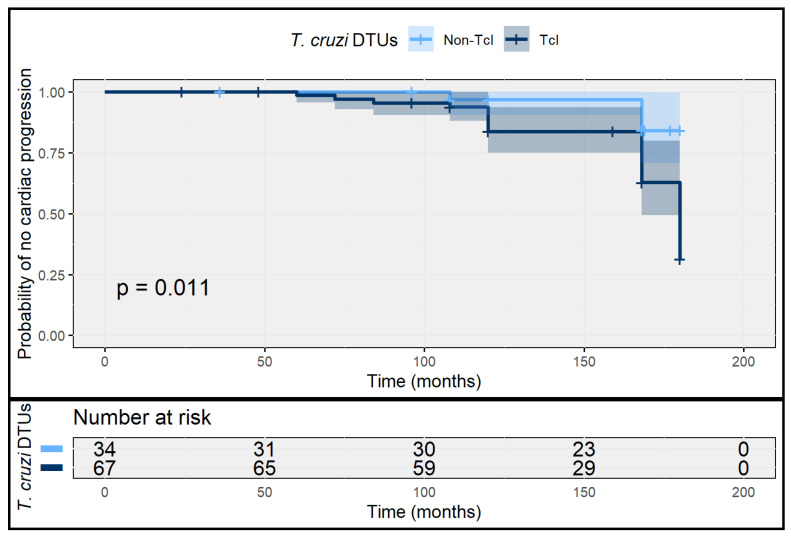
Time to cardiac progression after acute *T. cruzi* infection, stratified by DTU. Kaplan–Meier curves show the probability of remaining free of cardiac progression during follow-up among patients with acute *T. cruzi* infection, stratified by DTU TcI and non-TcI infections. Shaded areas represent 95% confidence intervals. Numbers at risk are shown below the plot.

The overall incidence rate of cardiac progression in the acute cohort was 2.04 cases per 100 person-years (95% CI 1.51–2.70). Stratified incidence rates were 2.61 per 100 person-years (95% CI 1.86–3.67) among TcI infections and 1.01 per 100 person-years (95% CI 0.52–1.92) among non-TcI infections. Time-to-event analysis showed earlier cardiac progression among TcI infections, with a shorter median time to progression (132 months; IQR 96–148) compared with non-TcI infections (168 months; IQR 132–180) (Figure 4).

In multivariable Cox proportional hazards analysis, DTU TcI infection, compared with non-TcI infections, was independently associated with cardiac progression (adjusted HR 3.1; 95% CI 1.7–5.6). Higher baseline parasitemia, older age, and prolonged residence in endemic areas were also independently associated with progression, whereas twelve or more years of formal education was associated with a lower risk (Table 2).

#### 3.4.2. Chronic Cohort

Among patients enrolled with indeterminate chronic infection, progression to chronic cardiac involvement occurred in 17 cases (14.3%) over a median follow-up of 182 months (IQR 120–216). The cumulative incidence of cardiac progression was statistically significantly higher among TcI infections (18.5%; 95% CI 11.6–28.7) than among non-TcI infections (5.3%; 95% CI 1.5–17.2).

**Figure 5:**
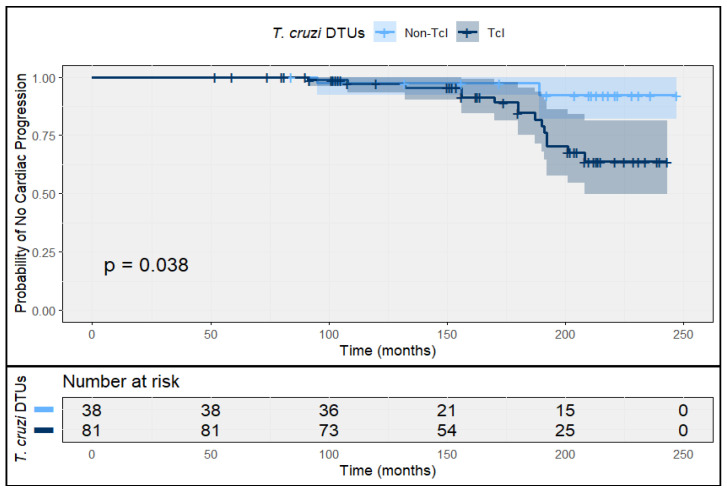
Time to cardiac progression in indeterminate chronic *T. cruzi* infection, stratified by DTU. Kaplan–Meier curves showing the probability of remaining free of cardiac progression among patients with indeterminate chronic T. cruzi infection, stratified by DTU TcI and non-TcI infections. Shaded areas represent 95% confidence intervals. Numbers at risk are shown below the plot.

The overall incidence rate was 1.05 cases per 100 person-years (95% CI 0.46–1.29), with stratified rates of 1.38 per 100 person-years (95% CI 0.57–1.73) for TcI infections and 0.38 per 100 person-years (95% CI 0.14–0.76) for non-TcI infections (Figure 5).

In multivariable Cox proportional hazards analysis, DTU TcI infection, male sex, presence of comorbidities, prolonged residence in endemic areas, and higher baseline parasitemia were independently associated with cardiac progression. In contrast, qPCR negativization at 12 months after treatment was independently associated with a lower subsequent risk of cardiac progression (Table 3).

### 3.5. Other Clinical Outcomes

In the acute cohort, three deaths (3.0%) and two composite cardiovascular events (2.0%) were recorded during follow-up. In the chronic cohort, nine deaths (7.6%) and seven composite cardiovascular events (5.9%) occurred. These events were recorded descriptively and were not included in the predefined secondary outcome of cardiac progression.

### 3.6. Sensitivity Analyses Accounting for Competing Risks

In sensitivity analyses treating death as a competing event, the association between DTU TcI infection and cardiac progression remained directionally consistent across clinical phases.

In the acute cohort, TcI infection was associated with a higher cumulative incidence of cardiac progression compared with non-TcI DTUs (Subdistribution HR 2.64; 95% CI 0.91–7.72), although this association did not reach conventional statistical significance (Gray test *p* = 0.063).

Similarly, in the indeterminate chronic cohort, TcI infection was associated with a higher cumulative incidence of cardiac progression (Subdistribution HR 3.59; 95% CI 0.81–16.0; Gray test *p* = 0.068).

These findings indicate that differential mortality did not account for the observed phase-specific differences in cardiac progression risk and support the robustness of the primary cause-specific Cox regression results.

### 3.7. Prognostic Value of Early qPCR Negativization

Across both clinical phases, qPCR negativization at 12 months after treatment was associated with a lower subsequent risk of cardiac progression in multivariable Cox models. This association was most pronounced in the indeterminate chronic cohort, where early qPCR negativity was associated with an approximately 80% reduction in the risk of cardiac progression (adjusted HR 0.20; 95% CI 0.10–0.50) (Table 3).

### 3.8. Serological Response After Treatment (Supportive Endpoint)

**Figure 6:**
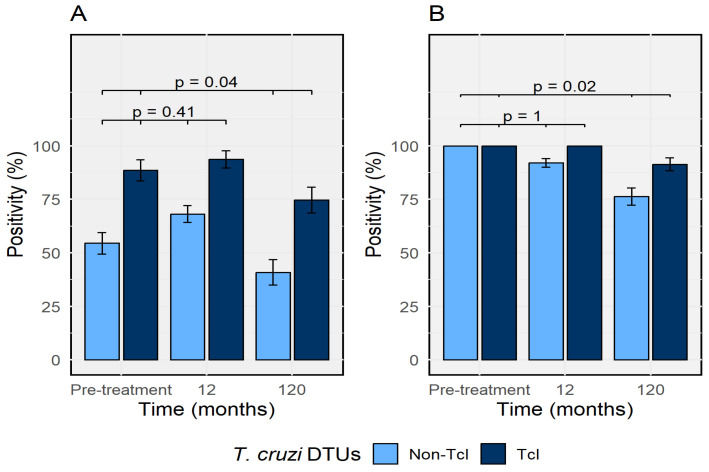
Serological response to etiological treatment in acute and indeterminate chronic *T. cruzi* infection. Panel (**A**) shows serological responses in acute infection, and Panel (**B**) shows serological responses in indeterminate chronic infection, stratified by DTU TcI and non-TcI infections. Bars represent antibody reactivity over time; error bars indicate standard errors.

Serological titers declined gradually during follow-up in both cohorts, with marked interindividual variability and frequent persistence of seropositivity despite sustained qPCR negativization. In the acute cohort, antibody declines were more pronounced among non-TcI infections than among TcI infections (Figure 6A). In the indeterminate chronic cohort, serological decline was slower overall and more delayed among TcI infections compared with non-TcI infections (Figure 6B).

## 4. Discussion

This cohort contributes to the ongoing debate on the clinical significance of DTU TcI in northern South America by integrating molecular, serological, and cardiac outcomes across disease phases. Instead of testing a deterministic DTU–phenotype model, this analysis evaluates how parasite genetic background interacts with exposure context, parasite burden, and host factors to shape long-term clinical trajectories. Previous evidence from Southern Cone countries has emphasized the severity of non-TcI lineages [14,15,16,17], whereas findings from TcI-predominant regions have been heterogeneous and strongly conditioned by ecological exposure [18,19].

In this context, three findings are particularly relevant. First, compared with non-TcI infections, TcI infection showed slower molecular clearance after therapy together with a higher burden of cardiac progression, a pattern better explained by differences in baseline parasite burden and cumulative exposure than by lineage-specific drug susceptibility or intrinsic virulence. Second, persistent seropositivity despite sustained qPCR negativization mirrors observations from other longitudinal cohorts and underscores the limited value of antibodies as short-term markers of parasitological cure, reflecting durable immune memory rather than ongoing parasitemia. Third, the strong association between early qPCR negativization at 12 months and lower subsequent risk of cardiac progression supports incorporating molecular monitoring into long-term risk stratification. Taken together, these findings favor a context-dependent interpretation of DTU-associated risk rather than a fixed DTU–phenotype relationship.

From a systems-oriented perspective, parasite genetic background operates within a broader ecological and biological framework. Transmission route, exposure intensity, baseline parasite burden, host characteristics, and structural determinants of health interact to shape long-term outcomes [27,28,29,30]. Accordingly, these findings contrast with earlier genotype-centered interpretations derived largely from Southern Cone settings, where DTUs TcII, TcV, and TcVI predominate and transmission ecologies differ substantially [16,17,31,32,33,34]. In TcI-predominant regions, clinical trajectories appear more tightly coupled to exposure context and disease phase than to parasite lineage alone [22]. Thus, TcI may be best interpreted as a marker of eco-epidemiological settings characterized by sustained transmission rather than as a direct causal determinant of adverse cardiac outcomes.

Differences in clearance kinetics between TcI and non-TcI infections reflected the rate of response rather than treatment failure, as sustained qPCR negativization was ultimately achieved in nearly all patients. Higher baseline parasitemia was a key correlate of delayed clearance, supporting the interpretation that early post-treatment qPCR positivity captures cumulative exposure intensity and parasite burden rather than intrinsic differences in drug susceptibility. In this study, qPCR was interpreted as a dynamic indicator of parasite burden and clearance kinetics rather than as an absolute marker of cure or therapeutic failure, particularly close to the assay’s limit of detection. These observations align with longitudinal studies documenting fluctuating low-level parasitemia close to molecular detection thresholds and underscore the limitations of binary post-treatment assessments in heterogeneous real-world settings [35,36,37].

Acute disease severity in this cohort was closely aligned with the epidemiology of oral transmission. Orally acquired infection has repeatedly been associated with intense early parasitemia and frequent myocardial involvement, reflecting high inoculum exposure rather than intrinsic parasite virulence [38,39]. The higher frequency of myocarditis observed among TcI infections should therefore be interpreted in light of regional parasite population structure and outbreak ecology [23,40]. In many TcI-predominant territories, severe acute presentations occur in outbreak settings consistent with oral transmission [20,41,42,43]. The clinical profile observed here mirrors patterns reported in other orally transmitted outbreaks, reinforcing the role of inoculum intensity and exposure context. This interpretation underscores the importance of addressing upstream drivers of infection through food safety, environmental surveillance, and outbreak prevention systems, rather than explanations centered solely on parasite genetics [38].

Long-term cardiac progression occurred across disease phases and was observed earlier and more frequently among TcI infections than among non-TcI infections; however, this pattern should not be construed as evidence of intrinsic TcI cardiotropism [9]. Risk clustered with markers of cumulative exposure and host susceptibility, including higher baseline parasitemia, prolonged residence in endemic areas, male sex, and comorbidities, reinforcing context-dependent interpretations of disease progression [13,44,45]. Within TcI, growing evidence highlights heterogeneity linked to domestic and sylvatic transmission cycles. TcISYL predominated among acute orally transmitted cases, whereas TcIDOM was more frequent in chronic infection, consistent with reports from Colombia [23,46]. These patterns support a continuum model in which clinical risk reflects the interaction between parasite population structure and exposure intensity rather than fixed lineage effects [47]. Widespread TcI circulation in sylvatic reservoirs further supports interpreting TcI as a marker of transmission context and cumulative exposure rather than intrinsic virulence [48]. Higher baseline parasitemia, used as a proxy for exposure intensity, was associated with slower molecular clearance and greater cardiac risk, reinforcing this context-dependent framework.

Serological trajectories further illustrate the multidimensional nature of post-treatment response. Persistent antibody positivity despite sustained molecular clearance was common, particularly in chronic infection, and reflects durable immune memory rather than ongoing parasitemia [49]. Slower serological decline among TcI infections may signal prolonged antigenic exposure or differences in host–parasite interaction, without diminishing the clinical relevance of molecular clearance [50]. Accordingly, molecular and serological markers should be viewed as complementary, addressing distinct biological processes, rather than as interchangeable indicators of therapeutic success [51,52,53,54].

Repeated exposure or reinfection in endemic settings may sustain immune stimulation and influence post-treatment trajectories. Experimental and clinical evidence indicates that recurrent exposure to *T. cruzi* can induce incomplete and strain-dependent protective immunity that modulates parasite burden without preventing reinfection [55,56,57]. Conversely, a classical Brazilian cohort from the 1970s showed that continuous exposure through repeated reinfections increased both the risk and severity of cardiomyopathy, underscoring that cumulative exposure can also be harmful depending on ecological and host context [58].

This non-binary model helps interpret the central observation of sustained qPCR negativization with persistent seropositivity, without assuming either definitive cure or ongoing high-grade parasitemia [59,60]. Intermittent low-level re-exposures may contribute to immune activation while not eliminating long-term cardiac susceptibility. Importantly, reinfection was not directly measured, and these interpretations remain hypothesis-generating rather than causal. Integrated studies incorporating exposure markers, strain tracking, and immunological profiling are required to determine whether post-treatment re-exposure confers functional protection or primarily reflects immunological memory.

These findings complement but differ from the BENEFIT trial, which enrolled patients with established Chagas cardiomyopathy and demonstrated that, despite improvements in parasitological markers, etiological therapy had limited impact on major clinical outcomes [53]. In contrast, the present results point to an earlier therapeutic window in TcI-predominant settings, where clearance kinetics captured at 12 months may still shape long-term trajectories. Early sustained qPCR negativization therefore emerges as a pragmatic prognostic marker, with potential to identify individuals who warrant intensified cardiological surveillance before structural myocardial damage becomes fixed [61].

Several limitations warrant consideration. The observational design limits causal inference, and residual confounding by unmeasured host, ecological, or social factors cannot be excluded. Grouping of non-TcI DTUs was necessary due to limited counts, precluding lineage-specific comparisons beyond TcI versus non-TcI. Selection based on detectable parasitemia resulted in a cohort enriched with higher parasite burden, which could affect early molecular clearance kinetics. Transmission route could not be reliably reconstructed in chronic infection, and molecular assays near the limit of detection are subject to variability. Importantly, sensitivity analyses accounting for death as a competing event yielded directionally consistent results across clinical phases. Although competing-risk models were underpowered to detect statistically significant differences due to the limited number of events, the persistence of higher cumulative incidence estimates among TcI infections indicates that differential mortality did not explain the observed associations with cardiac progression. These analyses support the robustness of the primary cause-specific Cox results and reinforce the interpretation of TcI-associated risk as reflecting disease dynamics rather than survival bias. While derived from a Colombian cohort, these findings are likely relevant to other TcI-predominant regions with similar ecologies; extrapolation to settings with different DTU distributions should be undertaken cautiously.

## 5. Conclusions

Sustained qPCR negativization at 12 months was associated with a substantially lower subsequent risk of cardiac progression in indeterminate chronic *T. cruzi* infection, supporting its use as a pragmatic prognostic marker. This phase-stratified cohort favors a context-informed interpretation of TcI-associated outcomes, in which delayed molecular clearance kinetics, persistent serological responses, and risk of cardiac progression reflect interactions among parasite genetics, transmission ecology, host factors, and structural conditions rather than deterministic lineage effects. Early sustained molecular response after treatment provides clinically meaningful information and may guide individualized follow-up and long-term surveillance. Moving beyond lineage labels toward context-aware risk stratification better captures the biological and clinical complexity of Chagas disease in TcI-predominant regions and could inform pragmatic, risk-adapted follow-up algorithms in these settings.

## Figures and Tables

**Table 1:** Clinical, epidemiological, and parasitological characteristics of patients with acute phase and indeterminate chronic *T. cruzi* infection.

Characteristic	*T. cruzi* Infection Status	
	Acute Phase (*n* = 101)	Chronic Phase (*n* = 119)
Age (years), median [IQR]	32 [20–49]	47 [41–51]
Male sex, *n* (%)	63 (62.8)	46 (38.7)
Systolic blood pressure (mmHg), mean ± SD	115 ± 10	130 ± 15
Heart rate (beats/min), mean ± SD	98 ± 15	65 ± 10
Body mass index (kg/m^2^), mean ± SD	24.5 ± 3.5	26.8 ± 4.2
Presence of *T. cruzi* DTU TcI, *n* (%)	79 (78.2)	81(68.1)
Median parasite load, (parasite equivalents/mL) [IQR]	6 [1–8]	2 [1–4]
Genotypes TcI		
TcI_SYL_, *n* (%)	65 (82.3)	11 (13.6)
TcI_DOM_, *n* (%)	14 (17.7)	70 (86.4)
Follow-up time (months), median [IQR]	168 [120–180]	182 [120–216]
Diabetes mellitus, *n* (%)	5 (4.9)	31 (26.1)
Systemic arterial hypertension, *n* (%)	10 (9.9)	47 (39.4)
Dyslipidemia, *n* (%)	8 (7.9)	34 (28.5)
Pre-existing heart disease (non-Chagasic), *n* (%)	6 (5.9)	39 (32.7)
Family history of Chagas disease *n* (%)	15 (14.8)	56 (47.1%)
Educational attainment (years) > 12, *n* (%)	41 (40.5)	45 (37.8)
>10 years in endemic area, *n* (%)	42 (41.6)	83 (69.7)
Blood transfusion	5 (4.9%)	10 (8.4)

DTU: Discrete Typing Unit; IQR: Interquartile Range; SD: Standard Deviation.

**Table 2:** Factors associated with cardiac progression after acute *T. cruzi* infection. Univariate and multivariable Cox proportional hazards models evaluating factors associated with cardiac progression among patients with acute *T. cruzi* infection. Hazard ratios (HRs) and 95% confidence intervals (CIs) are shown.

Covariates	Univariate Analysis			Multivariate Analysis		
	HR	95% CI	*p*-Value	HR	95% CI	*p*-Value
*T. cruzi* DTU						
Other	Ref					
TcI	2	1.2–3.5	0.008	3.1	1.7–5.6	<0.001
Time in endemic area						
<10 years	Ref					
≥10 years	2.4	1.4–4.0	0.001	2.1	1.2–3.7	0.014
Parasitemia	1.06	1.001–1.09	0.001	1.04	1.001–1.08	0.041
Age (years)	1.05	1.001–1.08	0.001	1.02	1.006–1.04	0.005
Years of education						
<12	Ref					
≥12	0.4	0.2–0.7	0.003	0.5	0.3–0.9	0.036

Ref: Reference.

**Table 3:** Factors associated with cardiac progression in indeterminate *T. cruzi* infection. Univariate and multivariable Cox proportional hazards models evaluating factors associated with cardiac progression among patients with indeterminate chronic *T. cruzi* infection. Hazard ratios (HRs) and 95% confidence intervals (CIs) are shown.

Covariate	Univariate Analysis			Multivariate Analysis		
	HR	95% CI	*p*-Value	HR	95% CI	*p*-Value
Sex						
Female	Ref					
Male	2.8	1.3–5.9	0.009	2.4	1.2–5.2	0.020
*T. cruzi* DTU						
Other	Ref					
TcI	2.6	1.2–5.5	0.012	2.3	1.1–5.1	0.038
Comorbidity						
None	Ref					
Yes	2.5	1.2–5.1	0.014	2.3	1.1–4.9	0.029
Time in endemic area						
<10 years	Ref					
≥10 years	2.4	1.1–5.0	0.018	2.2	1.0–4.7	0.041
Parasitemia	1.01	1.003–1.017	0.004	1.006	1.002–1.011	0.005
qPCR conversion at 12 m post-treatment						
No	Ref					
Yes	0.2	0.1–0.5	<0.001	0.2	0.1–0.5	<0.001

Ref: Reference.

## Data Availability

The data supporting the findings of this study are the property of the Instituto Nacional de Salud (INS) of Colombia and are subject to institutional data-sharing regulations. Access may be requested through the corresponding author, subject to reasonable request and INS approval.

## Abbreviations

The following abbreviations are used in this manuscript:

DTU	Discrete Typing Unit
qPCR	quantitative Polymerase Chain Reaction
ECG	Electrocardiograms
ELISA	Enzyme Linked Immunosorbent Assay
IFA	Indirect Immunofluorescence Assay
HR	Hazard ratios
IQR	Interquartile range
IC	Confidence intervals
